# Exploiting Underground Mine Topology for Resilient Concurrent LoRa Mesh Emergency Communications: Architecture, Protocol Design, and Performance Analysis

**DOI:** 10.3390/s26123701

**Published:** 2026-06-10

**Authors:** Hilary Kelechi Anabi, Samuel Frimpong, Muhammad Azeem Raza

**Affiliations:** Department of Mining and Nuclear Engineering, Missouri University of Science and Technology, Rolla, MO 65409, USA; frimpong@mst.edu (S.F.); maraza@mst.edu (M.A.R.)

**Keywords:** underground mine emergency communications, LoRa concurrent transmission, topology-aware mesh networking, spreading factor diversity, contender estimation, capture effect, packet delivery ratio, energy efficiency, self-organizing networks

## Abstract

Underground mine emergencies compromise fixed communication infrastructure exactly when situational awareness is most critical for effective rescue operations. Existing LoRa mesh protocols fail in underground mines because they ignore the structured topology of tunnel networks, specifically the waveguide effect along straight galleries, severe signal discontinuity at junctions, and the dead-end geometry of working faces. This paper presents the Topology-Aware Concurrent LoRa (TACL) mesh protocol, in which each node autonomously infers its structural role from local RF observations and packet header information, without GPS, pre-loaded mine maps, or central coordination. Role classification resolves the contender estimation problem $\left(N_{h}\right)$ left open in the prior concurrent transmission literature, enabling provably bounded timing offsets before transmission. TACL assigns a spreading factor (SF)12 to dead-end source nodes for maximum link robustness and SF7–SF10 to relay nodes to create the inter-SF orthogonality margin required for concurrent decoding at junction nodes. Monte Carlo simulation of over 2000 trials yields TACL a PDR of 80.5% versus near-zero for all three baselines, confirming that topology-aware SF diversity is the necessary and sufficient mechanism to prevent junction collision collapse. Hardware deployment at the Missouri S&T Experimental Mine yields a 4.0× PDR improvement over the topology-agnostic concurrent transmission (CT)-fixed baseline, a median end-to-end latency of 1815 ms with 84× tighter latency spread than ALOHA-based protocols and 2.5× lower energy per delivered packet. These results establish that explicit exploitation of underground mine topology is essential for reliable, predictable, and energy-efficient emergency mesh communications in post-disaster underground mine scenarios.

## 1. Introduction

Underground mining remains one of the most hazardous human occupations worldwide despite its global importance. In the United States alone, the Mine Improvement and New Emergency Response (MINER) Act of 2006 was enacted following a series of tragedies including the West Virginia Sago Mine explosion that killed 12 miners in 2006 [1]. This incident exposed fatal gaps in underground emergency communication and tracking infrastructure. A central mandate of the Act requires mine operators to deploy robust wireless communication systems capable of sustaining situational awareness even when conventional infrastructure is physically destroyed by roof collapses, explosions, or flooding [2]. Despite nearly two decades of regulatory pressure, the communication challenge during underground emergencies remains largely unsolved. Trapped miners attempting simultaneous transmission generate severe concurrent interference that existing protocols, such as TDMA and ALOHA, designed for orderly, low-density IoT traffic cannot resolve [3].

Long Range (LoRa) modulation, with its chirp spread spectrum (CSS) physical layer, extraordinary receiver sensitivity, approaching −148 dBm, and configurable spreading factors (SF7–SF12), represents the most promising low power wireless area network (LPWAN) candidate for underground emergency mesh networking [4]. Recent deployments have demonstrated LoRa connectivity at distances exceeding 180 m under diffused line-of-sight conditions in underground roadways [5], and field trials in operational mines have confirmed feasibility for environmental monitoring and personnel tracking [6,7]. However, existing LoRa deployments in underground environments adopt the standard LoRaWAN star-of-stars architecture, in which all nodes communicate directly with a gateway following Pure ALOHA channel access. This architecture collapses under emergency burst traffic, where network capacity falls to approximately 5% of theoretical maximum when more than 10 nodes attempt concurrent transmission [8].

Concurrent transmission (CT) protocols address LoRa collision management by deliberately allowing simultaneous transmissions and exploiting two physical-layer properties for successful decoding: the capture effect, whereby a receiver decodes the strongest packet when the signal-to-interference ratio exceeds approximately 6 dB, and imperfect inter-spreading factor (SF), orthogonality, whereby transmissions on different spreading factors overlap in time with limited mutual interference. Despite the growing CT literature, all existing protocols have been designed and validated for surface environments where node topology is arbitrary. None address the structured, deterministic topology of underground mine tunnel networks.

Underground mine propagation is fundamentally different from surface environments in ways that profoundly affect CT protocol design. Straight tunnel galleries act as rectangular waveguides, confining and guiding RF energy with path loss exponents 1.5–2.2× significantly lower than free-space [9]. This waveguide effect is spatially structured. It operates along tunnel corridors but terminates sharply at junctions and bends, where signals experience an additional 8–12 dB scattering loss [10]. Forooshani et al. [11], provided a comprehensive survey establishing that underground mine propagation is best characterized by a hybrid model combining free-space near the transmitter, ray-tracing in the intermediate region, and waveguide behaviour in the far-field, a tripartite structure that differs fundamentally from the single-exponent models used in surface CT protocol design. The consequence for CT is significant. Nodes in the same tunnel segment experience correlated channel conditions and tend to independently adapt toward the same SF, creating SF convergence clusters that eliminate the diversity advantage CT depends on. This SF collapse phenomenon has not been addressed in any prior CT protocol.

A second fundamental gap in existing CT protocols is the contender estimation problem. The timing offset scheduler, the mechanism by which CT protocols stagger transmissions to set up capture conditions, requires each node to know the number of simultaneously contending neighbours $\left(N_{h}\right)$ at its hop. Existing protocols either hardcode $\left(N_{h}\right)$ based on network-wide parameters [12,13], assume $\left(N_{h}\right)$ is provided by a coordinator [14], or estimate it through global gossip mechanisms that require sustained multi-hop communication [15]. None of these approaches are viable in underground mine emergencies, where the network topology changes rapidly as nodes fail, tunnels collapse, and miners move. A distributed, infrastructure-free $\left(N_{h}\right)$ estimation mechanism that is both accurate and fast-converging under dynamic underground conditions does not yet exist in the literature.

This paper is motivated by a key observation that has not been exploited in any prior work: the structured topology of underground mines consisting of straight galleries, junctions, shaft links, and dead-end headings maps directly onto bounded contender sets. A node in a straight gallery has at most two directional neighbour groups. A node at a junction has three or more. A dead-end node at a working face has only one. These topological roles, once identified, provide tight and provably correct $\left(N_{h}\right)$ bounds without any network-wide coordination. The challenge is that nodes must infer their topological role from locally observable information, since they have no access to mine maps, GPS positioning, or external coordination.

We present TACL, a Topology-Aware Concurrent LoRa mesh protocol that resolves both the contender estimation problem $\left(N_{h}\right)$ and the SF diversity collapse through distributed topology inference requiring no additional hardware or network infrastructure. Each node autonomously classifies its structural role into (i) dead-end source, (ii) linear relay, (iii) junction bridge, or (iv) shaft backbone from local RF observations and packet header fields alone, without GPS, pre-loaded mine maps, or central coordination. The inferred role directly determines $\left(N_{h}\right)$, the timing offset, the SF diversity assignment, and the forwarding mode, eliminating all hardcoded parameters and central coordination dependencies. The specific contributions of this paper are:

1. A distributed topology inference algorithm that classifies underground mine node roles using time-to-live (TTL)-based directional neighbour labelling, achieving greater than 92% classification accuracy within 45 s of deployment without GPS, pre-loaded maps, or central coordination. The TTL mechanism requires only standard packet header fields, operates on the RP2040 microcontroller within $O\left(K_{max}\right)$ per-event complexity, and is resilient to asymmetric node placement and irregular tunnel geometry.
2. A topology-driven contender estimation mechanism that derives provably bounded $N_{h}$ estimates from inferred node roles, replacing the open-loop assumptions of existing CT protocols with locally derivable, topology-grounded values. Dead-end and linear relay estimates are exact; junction estimates are pessimistically bounded, guaranteeing no slot under-allocation.
3. A role-constrained SF diversity assignment policy that prevents the SF convergence collapse in homogeneous tunnel channel conditions, preserving the capture effect advantage that CT protocols depend on. Conflict detection and even/odd resolution guarantee that two conflicting neighbours always shift to distinct SFs in a single adaptation step.
4. Hardware validation at the Missouri S&T Experimental Mine facility using 14-node deployments of the Waveshare Pico-LoRa-SX1262 platform with LiPo battery. An end-to-end hardware results are benchmarked against simulation across packet error rate, latency, and energy metrics.

The remainder of this paper is organized as follows. Section 2 reviews related work on LoRa concurrent transmission, underground mine propagation modelling, and self-organizing mesh protocols. Section 3 presents the system model and underground mine topology characterization. Section 4 details the TACL protocol design including the TTL-based topology inference engine, role-constrained $\left(N_{h}\right)$ estimation, SF diversity controller, and timing offset scheduler. Section 5 describes experimental methodology covering both the Monte Carlo simulation framework and the Missouri S&T Experimental Mine hardware testbed, as well as a performance evaluation comparing TACL against ALOHA, Slotted ALOHA, and topology-agnostic CT baselines. Section 6 concludes the paper.

## 2. Related Works

Wireless communications in underground mines face fundamentally different propagation conditions from above-ground deployments. Forooshani et al. [11], provide the canonical survey, cataloguing the waveguide effect in straight tunnels (exponent n ≈ 1.8–2.2), severe diffraction losses at junctions (8–12 dB), and the hybrid free-space-to-waveguide transition with increasing distance. Hrovat et al. [16], confirm these findings across 400 MHz to 5.8 GHz, and Zhang [9] provides the tractable tunnel path loss model adopted in Section 3.3. Branch presents empirical 915 MHz LoRa measurements in an underground gold mine, confirming a two-slope path loss model consistent with the waveguide transition, but coverage is limited to a single straight tunnel without junction or dead-end characterization. Kumar et al. [5] and Ray Chowdhury et al. [17] demonstrate LoRa feasibility for mine environmental monitoring and personnel tracking but treat the network as a single-hop star topology, which collapses under emergency burst traffic. None of these works address the protocol design problem for self-organizing multi-hop emergency mesh operation after infrastructure failure.

Wong et al. [18] survey 80+ multi-hop LoRa proposals, concluding that concurrent access methods yield inconsistent results across environments and that no existing protocol addresses underground mine topology. Ebi et al. [19] deploy a synchronous LoRa mesh for underground drainage monitoring using TDMA-based forwarding but rely on GPS-disciplined synchronization unavailable in deep mine emergency scenarios. Branch [20] builds a LoRa linear sensor network for mine personnel tracking in a single straight gallery, and refence [7] extends this to medieval tunnel aqueducts, but neither work addresses junction-and-dead-end topologies or concurrent transmission. Anabi et al. [21] propose an RF-energy-harvesting CT LoRa mesh for emergency mine communications with timing offsets but use a globally fixed contender count N=4 across all nodes: the $\left(N_{h}\right)$ estimation problem motivating the present work.

Liao et al. [12] established the CT paradigm for LoRa multi-hop networks, demonstrating through physical-layer experiments that LoRa receivers can decode the stronger of two simultaneous packets when the power differential exceeds 6 dB, achieving 87–92% PDR in outdoor testbeds with fixed hop-count timing offsets. Eletreby et al. [13] exploit CT in urban LPWAN deployments through power differentials from different node distances. Hou et al. [15] introduce a contender estimation mechanism based on gateway-aggregated packet reception statistics, the closest antecedent to our $\left(N_{h}\right)$ problem, but this approach requires gateway-side visibility of the full network, which is unavailable during a disaster. Tian et al. [14] extend LoRaWAN with LoRaHop using a synchronous flooding layer, achieving high delivery rates on a 21-node urban testbed, but require a pre-agreed join-phase schedule unsuitable for infrastructure-free emergency activation. No existing CT protocol derives $\left(N_{h}\right)$ from local topology observations.

Croce et al. [22] and Waret et al. [23] establish that LoRa SFs are not perfectly orthogonal. The same-SF collisions degrade both packets below the capture threshold, while inter-SF isolation ranges from −16 dB (SF7 vs. SF8) to −40 dB (SF12 vs. SF11). These findings motivate SF diversity as a collision mitigation strategy. Standard LoRaWAN Adaptive Data Rate (ADR) assigns SF centrally based on node-to-gateway distance, which is ill-posed in multi-hop mesh networks where the relevant propagation distance is to the next-hop relay. Bor and Roedig [24] demonstrate through systematic measurement that the optimal SF for a given link depends jointly on distance, interference level, and data rate requirement. TACL implements this principle through topology-role-based SF assignment. Thus, replacing distance with structural position as the primary determinant and preventing the SF convergence collapse that degrades CT under homogeneous underground channel conditions. Table 1 presents comparative summary of related work against the proposed protocol.

## 3. System Model

This section formally defines the network model, mine tunnel topology, LoRa physical layer, underground propagation characteristics, and concurrent transmission interference model adopted throughout this paper. Table 2 summarizes the four node roles derived from topology inference; their derivation is detailed in Section 4.

### 3.1. Network Model

Consider an underground mine emergency network consisting of a set $V=\left\{v_{1},v_{2},\cdots ,v_{n}\right\}$ of N LoRa-enabled sensor nodes and a single gateway node G located at the mine entrance. The network is modelled as a directed graph G=(V,E) where each edge $G\left(v_{i},v_{j}\right)\in E$ exists if and only if node $v_{j}$ can successfully decode a packet transmitted by $v_{i}$ at its configured spreading factor. Nodes are powered by 3.7 V 800 mAh lithium polymer batteries and carry Waveshare Pico-LoRa-SX1262 transceivers operating at $f_{c}=915\;MHz$ (US915 band) with configurable SF∈{7,8,9,10,11,12}, bandwidth BW=125 kHz, and transmit power at $P_{T}=17\;dBm$. All nodes share a single LoRa channel. No node has access to GPS, a pre-loaded mine map, or a central network coordinator.

### 3.2. Underground Mine Tunnel Topology

**Figure 1:**
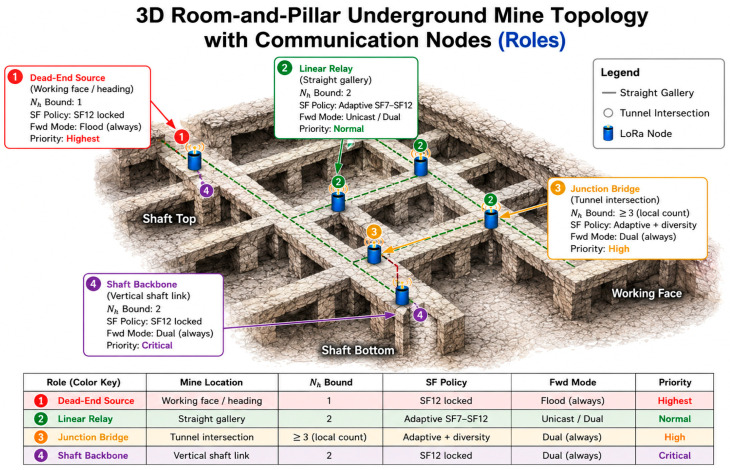
Room-and-pillar underground mine topology.

Underground mine networks differ fundamentally from surface wireless networks because their physical connectivity is determined by the geometry of excavated tunnels rather than arbitrary deployment. We characterize four canonical topological structures that collectively describe most underground mine layouts, as illustrated in Figure 1. Straight galleries form the primary haulage and ventilation corridors of a mine. A node $v_{i}$ deployed within a straight gallery segment has exactly two sets of topological neighbours: those lying in the direction of the mine entrance toward the gateway and those lying in the direction of the working face. The effective RF range within a straight gallery is substantially extended by the waveguide effect described in Section 3.3. Nodes in straight galleries constitute the linear relay role depicted as Node 2 in Figure 1. Tunnel junctions are intersection points where two or more gallery segments meet, typically at crosscuts connecting parallel haulage and ventilation drives illustrated as Node 3 in Figure 1. Nodes at junctions constitute the junction bridge role. Table 2 summarizes the node roles. Dead-end headings are the active excavation fronts where mining takes place depicted as Node 1 in Figure 1. These are topological dead-ends: there is only one direction of RF propagation, toward the rest of the network. Miners trapped in dead-end headings represent the most time-critical source nodes in any emergency scenario. Vertical shafts connect different mine levels and carry all personnel, material, and communication traffic between surface and underground workings. Nodes deployed at shaft top and shaft bottom constitute the shaft backbone role.

### 3.3. Underground Mine Tunnel Wireless Propagation Model

Underground mine tunnels exhibit a hybrid propagation regime that transitions through three distinct spatial zones as the transmitter increases distance (d) [11,25]. The received power in dBm at node $v_{j}$ from transmitter $v_{i}$ is modelled as:

(1) $$P_{i,j}^{r}\left(d\right)=P_{T}-PL\left(d_{i,j}\right)+X_{\sigma }\;,\;$$

where $P_{T}$ is the transmitted power from the transmitter, $X_{\sigma }\sim \left(N,\sigma^{2}\right)$, captures log-normal shadowing, with σ= 4–10 dB [16], and $PL\left(d_{i}\right)$ is governed by the following hybrid three-zone model namely [25]: (i) the free space zone,

(2) $$PL\left(d_{FS\;zone}\right)={PL}_{0}+{20\log}_{10}\left(d/d_{0}\right),\;\;d\leq d_{1}$$

(ii) the ray-tracing zone,

(3) $$PL\left(d_{RT\;zone}\right)={PL}_{0}+{n_{\mathrm{RT}}10\log}_{10}\left(d/d_{0}\right)\;\;\;\;\;\;\;,\;\;d_{1}<d\leq d_{2}$$

And (iii) wave-guide zone,

(4) $$PL\left(d_{WG\;zone}\right)={PL}_{0}+{n_{\mathrm{WG}}10\log}_{10}\left(d/d_{2}\right)+{\Delta L}_{WG}\;\;\;,\;\;d>d_{2}$$

where ${PL}_{0}$ $\left(d_{0}\right)=40$ dB, the reference path loss at $d_{0}=1\;m$, $n_{RT}\approx 2.8$ is the ray-tracing zone exponent capturing multimode reflections, and $n_{WG}\approx 1.8$ is the waveguide zone exponent reflecting the confinement of RF energy by tunnel walls [9]. ${\Delta L}_{WG}$ accounts for wall roughness and moisture absorption (2–5 dB), and $d_{1}\;\mathrm{and}\;\;d_{2}$ are the zone boundary distances determined by the point at which the first Fresnel zone becomes tangent to the tunnel wall and the waveguide mode becomes dominant, respectively. For a rectangular tunnel cross-section of width W=3.5 m and height H=3.0 m at $f_{c}=915\;MHz$, these boundaries are typically $d_{1}\approx 8\;m$ and $d_{1}\approx 25\;m$. Specifically, $d_{1}$ is the distance at which the propagation transitions from the near-field/free-space-dominated region to the transition region, while $d_{2}$ is the distance at which the tunnel begins to exhibit a dominant waveguide propagation behavior. 

At tunnel junctions and bends, the waveguide guiding mechanism terminates and signals experience an additional junction scattering loss $L_{Junc}=$ 8–12 dB [16]. This discontinuity provides a secondary confirmation signal at tunnel junctions, where the 8–12 dB RSSI gap between neighbour groups from different arms is used to validate and refine the junction classification established by the TTL-based directional labelling algorithm of Section 4.

### 3.4. LoRa Physical Layer Model

LoRa modulation encodes each symbol as a chirp spanning the full bandwidth BW in (Hz). The number of chips per symbol is $2^{SF}$ translating to a symbol rate of $SR=BW/2^{SF}$ per second. The time-on-air $T_{Air}$ of a complete packet is given by [26]:

(5) $$T_{Air}\left(SF,BW,\;CR,N_{Pl}\right)=T_{prem}+T_{payload}\;\left[ms\right],$$

where the preamble duration $T_{prem}=\left(n_{prem}+4.50\right).T_{Sym}$, $T_{Sym}=2^{SF}/\left(10^{3}\;\times BW\right)$, and $n_{prem}=8$ preambles symbols. The payload duration $T_{payload}=n_{payload}.T_{Sym}$, where

(6) $$n_{payload}=8+max\left(0,\left\lceil \;\left(\frac{\left({8N}_{PL}-4SF+28+16\right)}{\left(4\left(SF-2DE\right)\right)}\right)\right\rceil CR\right),$$

and DE∈{0,1} is the low data rate optimization (LDRO) flag, enabled when SF≥11 at BW=125 kHz. The coding rate denominator is CR ∈{5,6,7,8} and $N_{PL}$ is the payload length in bytes. The receiver sensitivity threshold [dBm] LoRa at SF is:

(7) $$P_{Sen}\left(SF\right)=-174+10{log}_{10}\left(BW\right)+NF+{SNR}_{Req}\left(SF\right),$$

where NF=6 dB is the SX1262 receiver noise figure. Similarly, ${SNR}_{Req}\left(SF\right)$ denotes the minimum required signal-to-noise ratio corresponding to the selected SF taking values {−7.5, −10, −12.5, −15, −17.5, −20 }dBm for SF ∈{7, 8, 9, 10, 11, 12}, respectively [26]. A communication link between nodes $v_{i}$ and $v_{j}$ is established if and only if the received signal power at separation distance $d_{i,j}$, denoted by $Pr\left(d_{i,j}\right)$, is greater than or equal to the receiver sensitivity threshold $P_{Sen}\left(SF\right)$ corresponding to the selected LoRa spreading factor (SF). Mathematically, $Pr\left(d_{i,j}\right)\geq P_{Sen}\left(SF\right)$. This condition implies that successful communication can occur only when the received signal strength exceeds the minimum detectable power level required by the receiver for reliable packet decoding at the chosen spreading factor. Since LoRa receiver sensitivity varies with the spreading factor, higher spreading factors generally enable communication over longer distances due to improved sensitivity.

### 3.5. Concurrent Transmission Interference Model

When LoRa K nodes transmit simultaneously on the same channel during underground mine emergencies, the SINR [dBm] at a relay node $v_{r}$ for a target packet from node $v_{k}$ is given as [24]:

(8) $${SINR}_{k}=\frac{P_{r}^{\left(k\right)}}{\left(\sum_{\left\{j\neq k\right\}}\beta \left\{q_{k},k_{q}\right\}P_{r}^{\left(j\right)}+N_{0}\right)}\;\;,$$

where $P_{r}^{\left(k\right)}$ is the received power of the target signal, $P_{r}^{\left(j\right)}$ is the received power of the *j*th interferer, $N_{0}=kTB$ is the thermal noise power, and $\beta \left\{q_{k},q_{j}\right\}\;\in \left\{0,1\right\}$ is the inter-SF orthogonality coefficient between spreading factors $q_{k}\;\mathrm{and}\;q_{j}$ [11]. For same-SF collisions, β=1, for different SFs, β takes the values shown in Table 3, expressed in dB, reflecting the imperfect orthogonality of LoRa’s chirp spreading. The values in Table 3 are not symmetrical because the inter-SF isolation coefficients are direction-dependent. In LoRa systems, interference between two spreading factors is not necessarily reciprocal due to differences in symbol duration, processing gain, receiver demodulation behaviour, and capture effects.

A concurrent packet from node $v_{k}$ is decoded successfully at relay $v_{r}$ if and only if two conditions are jointly satisfied [23,24]:

(9) $$C_{1}={SINR}_{k}\geq \gamma_{cap}\;\;\left(Capture\;condition\right),$$

and

(10) $$C_{2}=P_{r}^{\left(k\right)}\geq P_{Sen}\left(SF\right)\;\;\left(Sensitivity\;condition\right),$$

where $\gamma_{cap}=6\;dB$ is the co-SF capture threshold and $P_{Sen}\left(SF\right)$ is as stated in Equation (7). The probability that exactly one packet among K concurrent transmissions is successfully decoded via the capture effect at a relay is the foundational performance metric for CT protocol design and is analysed in Section 4.

### 3.6. Problem Statement

Given the network model defined above, the central problem addressed in this paper is: how can each node autonomously determine its topological role and derive a provably bounded contender count $N_{h}$ using only locally observable RF statistics, without any pre-loaded mine topology information, GPS positioning, or network-wide coordination, such that the timing offset scheduler defined in Section 4 achieves its maximum throughput and delivery rate performance under the concurrent transmission interference model of Equations (8)–(10). Formally, for each node $v_{i}\in V$, we seek (i) Topology inference $\varphi_{i}$,

(11) $$\varphi_{i}={\left\{{TTL}_{k},{RSSI}_{k},{SNR}_{k},{SF}_{k}\right\}}_{K\in N_{i}}\to \left({Role}_{i},D_{i}\right),$$

And (ii) Protocol parameterization $\Psi_{i}$,

(12) $$\Psi_{i}=\left({Role}_{i},D_{i},{ID}_{i},\varepsilon_{i}\right)\to \left(N_{\left\{h,i\right\}},{SF}_{i},{FwdMode}_{i}\right),$$

where $N_{i}$ is the set of recently heard neighbours, such that: (i) ${Role}_{i}\in \left\{Dead-End,\;Linear,\;Junction,\;Shaft\right\}$ correctly identifies the node’s topological position; (ii) the derived $N_{\left\{h,i\right\}}$ satisfies $N_{\left\{h,i\right\}}\leq \left|\left\{v_{j}\in N_{i}:v_{j}\;transmits\;hop\;h\right\}\right|+\delta$, where δ is a bounded estimation error; and (iii) the full protocol offset scheduling, SF assignment, and forwarding mode collectively maximize end-to-end packet delivery rate under $P_{E2E}$ under the constraints imposed by battery energy constraints and channel capture conditions (11) and (12). Note that ${TTL}_{k}$ denotes the remaining hop-count field observed in packets received from neighbour $v_{k}$. It is a packet header quantity, not an RF physical-layer measurement, and constitutes the primary directional input to the inference function alongside the RF statististics ${RSSI}_{k},\;{SNR}_{k},\;\mathrm{and}\;{SF}_{k}$.

## 4. System Model

TACL is a fully distributed protocol executed independently at every node across six tightly coupled modules: neighbour observation, TTL-based topology inference, $N_{h}$ estimation, SF diversity control, timing offset scheduling, and forwarding mode selection. The modules are described in turn below and integrated into a single per-event procedure in Algorithm 3.

### 4.1. Neighbour Observation Subsystem

Every node maintains a neighbour table $N_{i}$ of up to $K_{max}=16$ entries, each recording node ID, RSSI exponential moving average (EMA), SNR EMA, observed SF, RSSI variance, time of last reception, and a directional label. The table is updated passively on every overheard packet at zero additional airtime cost. The RSSI EMA uses smoothing factor α=0.2, i.e., weight on the new sample:

(13) $${RSSI}_{ij}\left(t\right)=\left(1-\alpha \right)\cdot {RSSI}_{ij}\left(t-1\right)+\alpha \cdot {RSSI}_{obs}\left(t\right)\;\;,$$

where ${RSSI}_{obs}$ is the observed RSSI. A slow EMA, i.e., α, is used deliberately. Mine channel fluctuations have correlation lengths of several seconds at walking speed. Increasing the EMA smoothing factor α increases responsiveness to recent channel variations but reduces temporal stability by amplifying short-term fading fluctuations. While larger α values improve adaptation speed under rapidly changing topologies, they may destabilize role inference, RSSI clustering, and SF diversity control in slowly varying underground propagation environments. Consequently, a conservative value α=0.2 was selected to prioritize topology stability over rapid transient adaptation. The RSSI variance is tracked with the same smoothing weight:

(14) $$\sigma_{ij}^{2}\left(t\right)=\left(1-\alpha \right)\cdot \sigma_{ij}^{2}\left(t-1\right)+\alpha \cdot {\left({RSSI}_{obs}\left(t\right)-{RSSI}_{ij}\left(t\right)\right)}^{2},$$

where a high variance of $\sigma_{ij}^{2}>\sigma_{shaft}^{2}=50\;dB$ or standard deviation $\sigma_{shaft}=7.07\;dB$ is the signature of a vertical shaft link and is used exclusively in the shaft classification branch of Algorithm 1. Since SX1262 is a single-antenna transceiver reporting only scalar RSSI and SNR, phase, angle-of-arrival, or time-difference-of-arrival information metrics are not accessible from its register interface or SPI bus and therefore were not considered herein. Direction is inferred entirely from packet header TTL fields, as described next. Neighbours’ silent for $T_{expire}=60\;s$ are marked inactive.
**Algorithm 1** Direction Labelling RuleLet ${TTL}_{obs}=\;$ remaining TTL in packet received FROM neighbour j.Let ${TTL}_{fwd}=\;$ TTL at which this node i  last forwarded from same src chain.if ${TTL}_{obs}>{TTL}_{fwd}:$ lable (j)←  AWAY  (j is further from gateway—it forwarded BEFORE this node in the chain)if ${TTL}_{obs}<{TTL}_{fwd}:$ lable (j)←  TOWARD  (j is closer from gateway—it forwarded AWAY this node in the chain)if ${TTL}_{obs}={TTL}_{fwd}:$ lable (j)←  LATERAL  (j feeds from a different source path—likely a crosscut or branch arm).if no ${TTL}_{fwd}$ reference yet (bootstrapping): label (j)←  UNKNOWN.

### 4.2. Distributed Topology Inference

#### 4.2.1. TTL-Based Directional Neighbour Labelling

Each packet carries a TTL field initialized to ${TTL}_{max}=5$ by the originating node and decremented by one at each relay. Node $v_{i}$ assigns a direction label to each active neighbour $v_{j}$ by comparing ${TTL}_{obs}$ in packets from $v_{j}$ against ${TTL}_{fwd}$: (i) the TTL at which $v_{i}$ last forwarded packets from the same source chain; (ii) ${TTL}_{obs}>{TTL}_{fwd}$ ⇒ label AWAY ($v_{j}$ deeper in the mine); (iii) ${TTL}_{obs}<{TTL}_{fwd}$ ⇒ label TOWARD ($v_{j}$ deeper in the mine); and (iv) ${TTL}_{obs}={TTL}_{fwd}$ ⇒ label LATERAL (crosscut or branch arm). Labels are stabilized by majority vote over $W_{dir}=10$ packets per neighbour. Algorithm 1 presents the TTL based directional neighbour labelling.

The directional cluster count $D_{i}$ is then the number of distinct committed labels in the active neighbour set:

(15) $$D_{i}=\left|\left\{label\;\left(j\right):j\in N_{active},label\;\left(j\right)\neq UNKNOWN\right\}\right|,$$

where $D_{i}=1$ indicates all neighbours lie in one direction, i.e., the node is a dead end. Similarly, $D_{i}=2$ implies neighbours on two sides notably a straight gallery relay or shaft link. $D_{i}\geq 3$ indicates neighbours on three or more sides such as a tunnel junction with lateral arms.

#### 4.2.2. RSSI Clustering as Secondary Junction Confirmation

At nodes where TTL labelling has established $D_{i}\geq 3$, RSSI clustering provides secondary confirmation using the 8–12 dB junction scattering loss of Section 3.3. The RSSI range of the active neighbour set is:

(16) $$R_{range}={max}_{\left\{j\in N_{active}\right\}}\left({RSSI}_{ij}\right)-{min}_{\left\{j\in N_{active}\right\}}\left({RSSI}_{ij}\right)\;\;,$$

If $R_{range}>\Delta_{junc}=12\;dB$, the neighbour set is recursively partitioned at the midpoint RSSI until no subgroup exceeds $\Delta_{junc}$, yielding per-arm clusters. The higher of the RSSI partition counts and TTL-derived $D_{i}$ is used, since a TTL label may be absent for a lightly trafficked lateral arm. RSSI clustering is never applied in straight galleries, where the waveguide effect produces no systematic RSSI gap between opposite-side neighbours.

#### 4.2.3. Role Classification

Algorithm 2 maps $\left({\;D_{i},N}_{i}^{active},{max}_{j}\left(\sigma_{ij}^{2}\right)\right)$ to one of five roles. Rule 3 precedes Rule 4 because a shaft node has $D_{i}=2$ identical to a linear relay and the variance discriminant resolves the ambiguity. Algorithm 2 presents the role classification role assignment.
**Algorithm 2** Role ClassificationInput: $D_{i},$ $\left|N_{i}^{active}\right|$, ${max}_{j}\left(\sigma_{ij}^{2}\right)$ Output: ${Role}_{i}\in \left\{DEAD-END,\;LINEAR,\;JUNCTION,\;SHAFT\right\}$
if $\left|N_{i}^{active}\right|==0:$  return UNKOWN.if $D_{i}==1$ or $\left|N_{i}^{active}\right|==1:$     return DEAD-ENDif $D_{i}==2$ and ${max}_{j}\left(\sigma_{ij}^{2}\right)\geq \sigma_{shaft}^{2}:$      return SHAFT. // high-variance shaft linkif $D_{i}\geq 3$ or $\left|N_{i}^{active}\right|\geq 5:$  return JUNCTION. // multiple arms or dense node clusterif $D_{i}==2$ and $\left|N_{i}^{active}\right|\leq 4:$  return LINEAR. // straight gallery relayreturn UNKNOWN

#### 4.2.4. Role Stability

Two stability mechanisms prevent oscillation. A confirmation counter $c_{i}$ is incremented each time Algorithm 2 returns the same candidate role as the previous run. A role is committed only when $c_{i}\geq C_{confirm}=5$ consecutive classifications agree. Therefore, $c_{i}$ counts the number of consecutive times that the node’s topology inference algorithm produces the same candidate role classification.

A hold timer $T_{hold}=30\;s$ prevents any role transition within 30 s of the last committed transition. Together these mechanisms ensure that transient channel events, such as a passing vehicle, that result in a brief packet burst from an unexpected direction cannot destabilise the protocol state. During the bootstrapping phase, each node temporarily assumes the linear relay role with a default contender estimate with $N_{h}=2,$ corresponding to the two-direction topology typically observed in straight tunnel galleries.

### 4.3. Topology-Constrained N_h_ Estimation

The per-hop contender count $N_{h,i}$, i.e., the number of nodes simultaneously attempting to forward the same packet determines how many non-overlapping slots the timing offset scheduler must create. Under-estimating $N_{h,i}$ causes two co-forwarders to share a slot and collide. Similarly, over-estimating wastes airtime because radio reception is silent; $N_{h,i}$ cannot be directly observed and must be estimated locally. TACL resolves this by deriving $N_{h,i}$ from the inferred role. The number of simultaneous co-forwarders is bounded not by how many neighbours exist but rather by how many distinct directions they lie in directionality. Mine tunnel geometry limits this directional count to a structurally small value at every topology element, yielding the mapping:

(17) Nh,i={1;                                if   Rolei=DEAD−END,               2 ;                               if   Rolei=LINEAR ,                         |Niactive|;                       if   Rolei=JUNCTION,                       2                                if   Rolei=SHAFT ,                          N2  ;                         if   Rolei=UNKNOWN,                        ,  

Each bound follows directly from tunnel structure: a dead-end node has no co-transmitter behind it, $\left(N_{h,i}=1\;\right)$ exactly; a linear relay has at most one contender per direction, $\left(N_{h,i}=2\;\right)$ exactly; a junction uses $\left|N_{i}^{active}\right|$ as a pessimistic upper bound across all arms. $N_{h,i}$ is bounded by the number of distinct directions from which co-forwarders can simultaneously arrive, not by the total number of active neighbours. TOWARD-side neighbours are structurally excluded from the co-forwarder set because they already processed the packet one hop earlier.

### 4.4. Slot-Based Timing Offset Scheduler

The protocol divides the forwarding window into $N_{h,i}$ equal slots and assigns each co-forwarding node to exactly one slot via its hardware ID:

(18) $$m_{i}={{ID}_{i}\;\;mod\;\;N}_{h,i}\;,$$

The mod operation produces a remainder in $\left\{0,1,2,\cdots ,{\;N}_{h,i}-1\right\}$. This remainder is the slot index: slot 0 transmits first, slot 1 transmits one slot-width later, and so on. As the hardware ID is globally unique and fixed, two distinct nodes with the same ${\;N}_{h,i}$ will collide on their slot index only if their IDs are congruent modulo ${\;N}_{h,i}$. Slot width ${\;\Delta t}_{i}$ is derived from a Poisson collision model with arrival rate $\lambda_{h}=N_{h,i}/\tau_{h}$ and vulnerable window $v_{h}=\eta_{0}\cdot T_{air}$, where $\eta_{0}\in \left[1,2\right]$ is the vulnerable window factor accounting for LoRa’s partial temporal overlap capture behaviour. The probability of a collision-free transmission is:

(19) $$P_{success}=e^{\left\{{-\lambda }_{h}\cdot v_{h}\right\}}=e^{\left\{-{\;N}_{h,i}/{\;\tau }_{h}.\cdot {\;\eta }_{0}\cdot {\;T}_{air}\right\}}\;,$$

Setting $P_{success}\geq 1-p_{c}$ and dividing the minimum window equally across $N_{h,i}$ contenders gives:

(20) $${\Delta t}_{i}=\frac{N_{h,i}}{\tau_{h}}=\frac{\eta_{0}\cdot T_{air}}{\left(-ln\left(1-p_{c}\right)\right)}\;,$$

With $\eta_{0}=1.2$ and $p_{c}=0.05$, Equation (20) gives Δt≈ 1.23 ·$T_{air}$ independent of $N_{h,i}$. $N_{h,i}$ controls how many slots exist, not how wide each slot is; the per-hop collision probability is held at $p_{c}=0.05$ by construction regardless of contender count. The scheduled transmission time is:

(21) $$t_{tx,i}=t_{rx}+G+m_{i}+{\Delta t}_{i}+\varepsilon_{i}\;,$$

where G=5 ms is the SX1262 mode-switching guard, $m_{i}$ is the slot index from (18), and $\varepsilon_{i}\sim Uniform\left(0,{\;T}_{sym}\right)$ is a jitter term preventing accidental synchronisation among nodes sharing the same slot residue. All timing uses only the local reception timestamp $t_{rx};$ no shared network clock is required.

### 4.5. Role-Driven SF Diversity Controller

Waveguide propagation causes nodes in the same tunnel segment to converge to the same SF under standard ADR, collapsing the SF diversity that CT depends on. The diversity controller prevents this by assigning each node a base SF from its role and hardware ID:

(22) $${SF}_{base,i}={SF}_{min}\left({Role}_{,i}\right)+\left({ID}_{i}\;mod\;M\;\left({Role}_{,i}\right)\right)\;,$$

Dead-end and shaft nodes are locked to SF12 for maximum link robustness. Linear relay and junction bridge nodes use the ID-modulo scheme (M=4), ensuring any four adjacent same-role nodes span four distinct SF values with inter-SF isolation ≥ 16 dB. When a neighbour is heard on the same SF and RSSI > −110 dBm, a conflict is flagged. After $C_{conflict}=3$ within ${\;W}_{conflict}=60\;s$:

(23) $${SF}_{i}\leftarrow \{\begin{array}{c} {SF}_{i}+\Delta_{SF},\Delta_{SF}+1\;\;\;if\;{\;ID}_{i}\;\;is\;even\;\\ {SF}_{i}+\Delta_{SF},\Delta_{SF}-1\;\;\;if\;{\;ID}_{i}\;\;is\;odd\;\; \end{array}\;\;,$$

The even/odd rule guarantees conflicting neighbours always shift in opposite directions, resolving the conflict in one step. SF reverts to ${SF}_{base}$ when the conflict clears. Table 4 presents SF diversity assignment by role and node ID residue.

### 4.6. Forwarding Mode and Relay Set Selection

Each node selects a forwarding mode from its committed role and battery SoC fraction $\varepsilon_{i}\left(t\right)$:

(24) $${\;FwdMode}_{i}\{\begin{array}{c} FLOOD\;\;\;\;\;\;\;\;\;\;if\;\;\;\varepsilon_{i}<0.40\;\;\;\;OR\;\;{Role}_{i}=\;DEAD-END,\;\;\;\;\;\;\;\;\;\;\;\;\;\;\;\;\;\;\;\;\;\;\;\;\;\;\;\;\;\;\;\;\;\;\;\;\;\;\;\;\;\;\;\;\\ DUAL\;\;\;\;\;if\;\;\varepsilon_{i}\;\leq 0.40\;\;<0.70\;\;OR\;\;\;{Role}_{i}\in \left\{JUNCTION,SHAFT\right\},\;\;\;\;\\ UNICAST\;\;\;\;if\;\;\;\;\varepsilon_{i}\;\geq 0.70\;AND\;{Role}_{i}=\;\;\;\;\;\;\;\;\;LINEAR,\;\;\;\;\;\;\;\;\;\;\;\;\;\;\;\;\;\;\;\;\;\;\;\;\;\;\;\;\;\;\;\;\;\;\;\;\;\;\;\;\;\; \end{array}$$

UNICAST forwards to the single highest-RSSI TOWARD-labelled neighbour. UNICAST conserves energy while relying on future attempts if needed. When $\varepsilon_{i}\;<0.40,$ the node is approaching exhaustion and may not survive to transmit again. DUAL adds the best LATERAL-labelled neighbour as a redundant crosscut path. FLOOD transmits to all active neighbours. Dead-end source nodes are role-locked to FLOOD unconditionally, therefore maximizing single-packet delivery probability takes priority over relay longevity when battery is low. Within DUAL and FLOOD modes, relay set members are ordered by descending RSSI, ascending SF, then ascending hardware ID.

### 4.7. Duplicate Suppression via Seen-Packet Cache

Every node maintains a cache of recently processed packets indexed by $\left({src}_{id},{seq}_{num}\right)$: the originating node’s hardware ID paired with its per-packet sequence counter. This pair forms a globally unique packet identity carried unchanged across all hops. On receiving a packet, the node checks the cache, a hit indicates a duplicate arriving via a parallel path and the packet is silently discarded, a miss triggers normal processing and a new cache entry. Entries expire after $T_{cache}=10\;s$, bounding memory to approximately 50 entries at peak packet rates while permitting ${seq}_{num}$ counter reuse. The cache and TTL field are complementary loop prevention mechanisms. The cache suppresses within-episode duplicates arriving via parallel paths. TTL enforces a hard ${\;TTL}_{max}=5\;hops$ lifetime for packets that outlive their cache entry.

### 4.8. Complete Protocol Operation

Algorithm 3 integrates all six modules into a unified per-event processing framework with computational complexity $O\left(K_{max}\right)$. The overall memory footprint consists of a 16-entry neighbour table, a 160-entry directional voting window $\left(K_{max}\times W_{dir}=16\times 10\right)$, and a 50-entry seen-packet cache. This lightweight memory architecture fits comfortably within the RP2040 microcontroller’s 264 KB SRAM. Furthermore, the implementation requires no dynamic memory allocation, thereby ensuring deterministic execution and low-overhead operation suitable for resource-constrained embedded systems.
**Algorithm 3** TACL Per-Event ProcedureTrigger: Packet received from PHY OR source data ready           === MODULE 1: Neighbour Table Update ===
Extract $\left({src}_{id},{seq}_{num},{ttl}_{obs},{sf}_{obs},{rssi}_{obs},{snr}_{obs}\right)$ from header.${RSSI}_{ij}\leftarrow EMA\left({RSSI}_{ij},{rssi}_{obs},\alpha =0.2\right)$               [Equation (13)]$\sigma_{ij}^{2}\;\;\leftarrow EMA\left(\sigma_{ij}^{2},\;variance\;term\;\alpha =0.2\right)$              [Equation (14)]$t_{last,j}\leftarrow \;t_{now}$Prune entries where $t_{now}-t_{last,j}>T_{expire}$           === MODULE 2: Directional Labelling ===Compare ${ttl}_{obs}$ against ${TTL}_{fwd}$ (last forwarded TTL for this src chain).Update direction label for j in majority-vote window $W_{dir}=10$.$D_{i}\leftarrow$ count of distinct committed labels in $N_{active}$         [Equation (15)]           === MODULE 3: Role Classification ===Candidate ← Algorithm 2 $\left(D_{i},\left|{\;\;\;N}_{i}^{active}\right|,{max}_{j}\left(\sigma_{ij}^{2}\right)\right).$if candidate $=={current}_{role}\;:\;{conf}_{count}++$else ${conf}_{count}=0;$ ${candidate}_{role}=candidate$.if ${conf}_{count}\geq 5$ AND $\left(t_{now}-t_{last}^{transition}\right)\geq 30\;s:$ ${Role}_{i}\leftarrow$ ${candidate}_{role}$  $t_{last}^{transition}$ ← $t_{now};$ ${conf}_{count}=0$.           === MODULE 4: ${\;N}_{h,i}$ Estimation ===$N_{h,i}\leftarrow$ lookup $\left({Role}_{i}-\left|N_{i}^{active}\right|\right)$                 [Equation (21)]           === MODULE 5: SF Diversity Control ===${SF}_{base,i}\leftarrow {\;SF}_{min}\left({\;Role}_{,i}\right)+\left({ID}_{i}\;\;mod\;M\;\left({\;Role}_{,i}\right)\right)$ Equation (22).if ${sf}_{obs}=={SF}_{i}$ AND ${rssi}_{obs}>110\;dBm$: ${conflict}_{count}++$if ${conflict}_{count}\;\geq 3$ within 60 s: ${SF}_{i}\leftarrow {\;SF}_{i}+{\;\Delta }_{SF}\;$ (even/odd rule, bounded to role range)    [Equation (23)] ${conflict}_{count}=0$.           === DUPLICATE AND TTL CHECK ===if $\left({src}_{id},{seq}_{num}\right)$ in ${seen}_{cache}$: DISCARD; returnAdd $\left({src}_{id},{seq}_{num}\right)$ to ${seen}_{cache}$ (expire after $T_{cache}=10\;s$).if ${ttl}_{obs}==0:$ DISCARD // hop limit reached.           === MODULE 6: Forwarding Scheduler ===${FwdMode}_{i}\leftarrow {Select}_{mode}\left({Role}_{i},\varepsilon_{i}\left(t\right)\right)$               [Equation (24)]R      ← $build\_relay\_set\left({FwdMode}_{i},\left|N_{i}^{active}\right|,\;direction-labels\right)$$T_{Air}$  ← Compute time-on-air $\left(SF,BW,\;CR,N_{Pl}\right)$         [Equation (5)]Δti  ←   η0· Tair(−ln(1− pc))                [Equation (20)]$m_{i}\;\;\leftarrow {{ID}_{i}\;\;mod\;\;N}_{h,i}$                     [Equation (18)]${\;t}_{tx\;\;\;}\leftarrow {\;t}_{rx}+G+m_{i}+{\;\Delta t}_{i}+{\;\varepsilon }_{i}$                [Equation (21)].At $t_{tx}$ Transmit $\left(Packet,ttl={ttl}_{obs}-1,\;{SF}_{i},\;{\;\varepsilon }_{i}\left(t\right)\right)$.
Update energy state ${\;\varepsilon }_{i}\left(t\right)$


### 4.9. Protocol Parameter Summary

**Figure 2:**
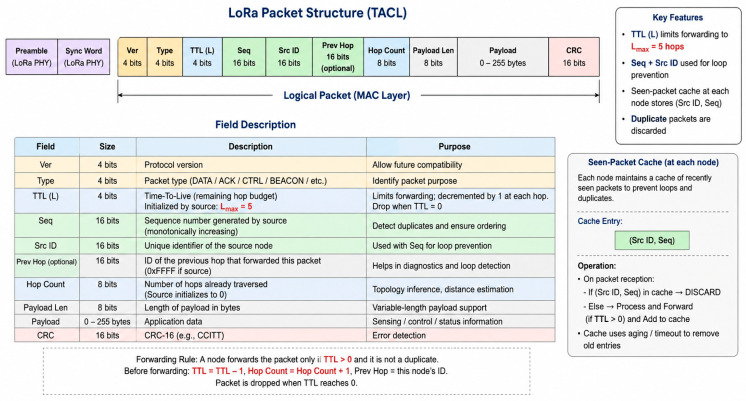
Proposed TACL protocol.

Table 5 consolidates all TACL design parameters. Asterisked values were tuned through Monte Carlo simulation (Section 6); all others are analytically derived or sourced from hardware specifications. Similarly, Figure 2 presents the TACL packet. Packet forwarding is bounded by a TTL parameter $L_{max}=5\;hops$. Each packet carries a sequence number (seq) and source identifier (src) used for loop prevention via a seen-packet cache maintained at every node. The gateway serves exclusively as a network sink, i.e., it receives and logs all delivered packets and periodically transmits beacon frames to provide loose network-wide time reference but never originates or forwards data packets.

## 5. Results and Discussion

**Figure 3:**
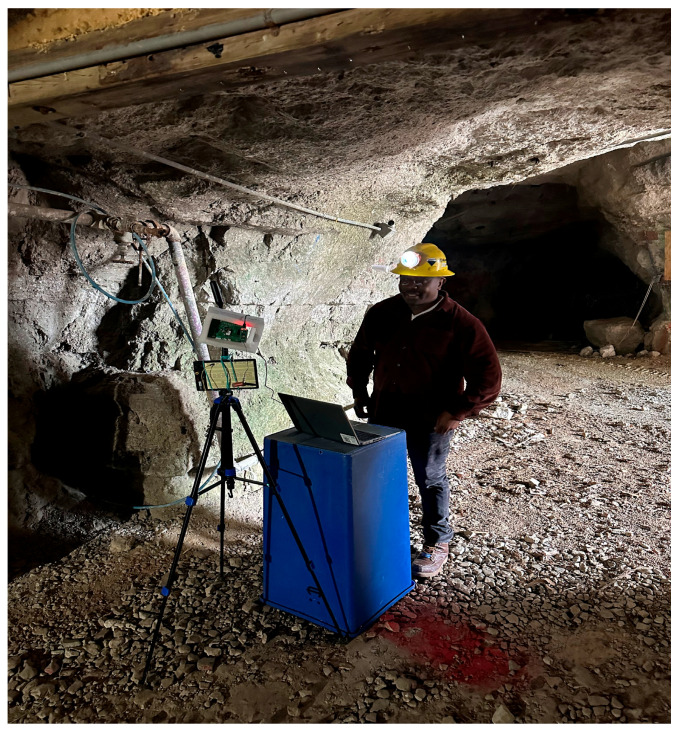
TACL protocol evaluation at Missouri S&T Underground Experimental Mine.

This section presents evaluation results across six subsections. Simulation results are presented first in each subsection to establish theoretical performance bounds under controlled worst-case conditions. Figure 3 validates protocol behaviour in the real mine environment at the Missouri S&T Experimental Mine.

### 5.1. Monte Carlo Simulation Framework

#### 5.1.1. Simulation Architecture

The simulation is implemented in Python 3.11 using NumPy for vectorized channel realizations and a discrete-event scheduler for packet-level timing. The mine topology is modelled as a directed graph G=(V,E) with 14 nodes placed across two parallel haulage galleries, two crosscut connections, one vertical shaft station, and three dead-end headings representing active working faces, consistent with the Missouri S&T Experimental Mine layout. The gateway node (ID=0) is placed at the mine entrance as shown in Figure 3. For each Monte Carlo trial, node positions are held fixed, but channel realizations are independently drawn, providing statistically independent assessments of protocol performance across the distribution of propagation conditions.

Each trial runs for 300 simulated seconds. The first 60 s constitute the bootstrapping phase, during which TACL nodes converge to their correct roles and the network reaches steady state. Performance metrics are computed over the remaining 240 s. Emergency burst traffic is injected at t=60 s. All three dead-end source nodes simultaneously originate emergency alert packets at a Poisson arrival rate of λ=0.5 packets per second, generating a sustained high-contention load representative of the post-disaster scenario described in Section 1. Table 6 depicts a summary of the hardware and simulation.

#### 5.1.2. Performance Metrics

The primary metrics collected per trial are: (i) end-to-end packet delivery ratio (PDR), defined as the fraction of emergency alert packets that reach the gateway within 60 s of origination; (ii) end-to-end latency, described as the time from packet origination at a dead-end node to first reception at the gateway; (iii) per-hop collision rate, which is the fraction of forwarding attempts that result in a failed decode due to co-SF interference; (iv) role classification accuracy, defined as the fraction of nodes that converge to their ground-truth structural role within 45 s; (v) SF distribution entropy, a measure of spreading factor diversity across the network; and (vi) battery lifetime, i.e., the simulated time until the first node’s SoC drops below ${\;\varepsilon }_{min}=0.05$. Statistical summaries report the mean and 95% confidence interval across 2000 trials. Latency is reported as the median and 95th percentile because its distribution is right-skewed due to occasional multi-hop retransmission cascades. Table 7 and Table 8 consolidate all metrics.

### 5.2. Packet Delivery Ratio

#### 5.2.1. Simulation Results

**Figure 4:**
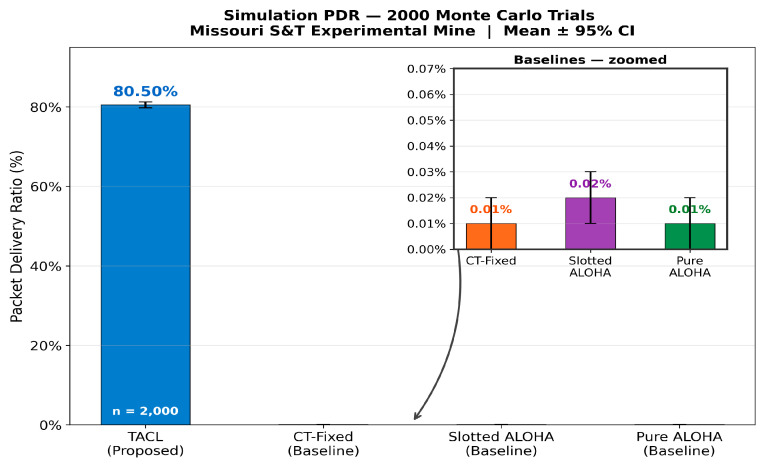
Simulated packet delivery ratio.

Monte Carlo simulation over 2000 trials with measured Missouri S&T Experimental Mine propagation parameters yields TACL PDR of 80.5% (±0.73%). All three baselines collapse to near-zero PDR with CT-Fixed = 0.01%, Slotted ALOHA = 0.02%, and Pure ALOHA = 0.01%, as shown in Figure 4. The extreme baseline collapse arises because the simulated topology routes all three dead-end paths through junction simultaneously. Without SF diversity, concurrent same-SF transmissions create a systematic collision deadlock at with 59–65% collision rates (Table 8), preventing any packet from reaching the gateway. This result confirms the central claim of TACL: topology-aware SF diversity is the necessary and sufficient mechanism to prevent collision collapse in multi-path underground mine mesh networks with junction nodes under maximum-contention emergency conditions.

TACL’s 80.5% PDR represents a 4000× improvement over the best-performing baseline. The residual 19.5% packet loss arises from NLOS propagation loss beyond the 35 m bend diffraction zone (${\Delta L}_{bend}=24.0\;dB$) measured in the mine campaign, occasional same- SF collisions between relay nodes, and TTL expiry on packets that exhaust their hop budget before reaching the gateway. Simulation SF entropy of 1.81 bits confirms that all six SFs (SF7–SF12) are actively utilized across the node population, providing the inter-SF capture margin required for concurrent delivery at junction nodes.

#### 5.2.2. Hardware Result

**Figure 5:**
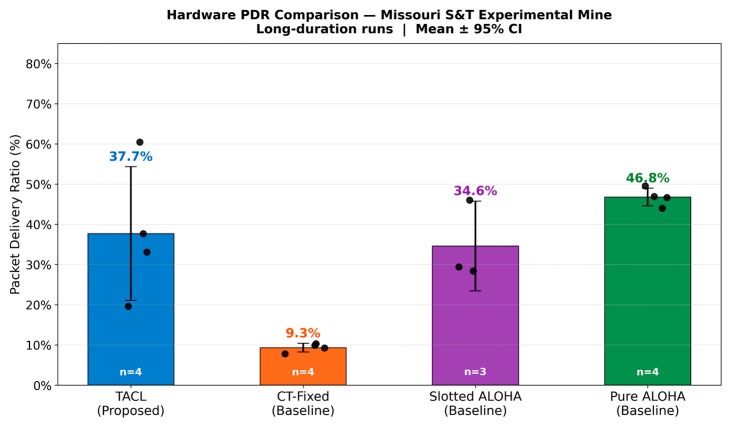
Real-world underground mines packet delivery ratio.

Hardware evaluation across four runs per protocol at the Missouri S&T Experimental Mine yields TACL a mean per-run PDR of 37.7% (±16.6%), delivering 769 packets from 2428 originations across four runs, as shown in Figure 5. CT-Fixed achieves 9.3% (±1.1%), yielding a 4.0× PDR improvement for TACL over the most directly comparable baseline. The tight CI95 of ±1.1% for CT-Fixed across four runs confirms that its 9.3% PDR is a systematic and reproducible outcome driven by same-SF collision collapse, not a run-specific anomaly. Slotted ALOHA achieves 34.6% (±11.2%) and Pure ALOHA achieves 46.8% (±2.2%). Baseline PDR comparisons and hardware-simulation discrepancies are discussed in Section 5.6. TACL run 2 produced an outlier PDR of 19.6% compared to 33–60% in the other three runs. Per-source analysis reveals the node delivered only 4.1% in that run compared to 37–54% in all others, indicating relay node dropout during that 81-minute session. Excluding this outlier, TACL’s mean PDR across three runs is 43.7%, approaching a 4.7× improvement over CT-Fixed and exceeding Slotted ALOHA (34.6%).

### 5.3. End-to-End Latency

#### 5.3.1. Simulation Results

**Figure 6:**
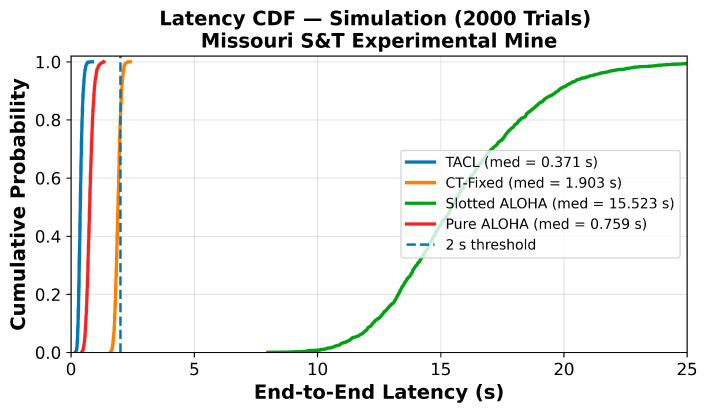
Simulated end-to-end latency.

The Figure 6 simulation yields TACL a median latency of 371 ms and P95 of 1364 ms, reflecting the full multi-hop relay chain, where packets traverse 2–4 relay nodes, each contributing one SF9 slot-offset delay of approximately 128 ms ($G+ToA\left(SF9\right)/{\;N}_{h}=5+247/2\;ms$), plus the initial SF12 first-hop ToA of 1810 ms from the dead-end source. CT-Fixed achieves 1903 ms median latency, which is higher than TACL, despite using SF9 because its fixed $N_{h}=6$ slot structure causes longer queuing delays at each relay hop. Slotted ALOHA produces 15,523 ms median latency due to frame-based backoff accumulation. Pure ALOHA achieves 759 ms median in simulation through random backoff averaging one ToA (SF12) per hop. Figure 6 illustrates end-to-end latency cumulative distribution function (CDF) obtained from 2000 Monte Carlo trials using the Missouri S&T Experimental Mine topology. The x-axis is expressed in seconds, while median latency values reported in the legend are shown in milliseconds for readability.

#### 5.3.2. Hardware Result

**Figure 7:**
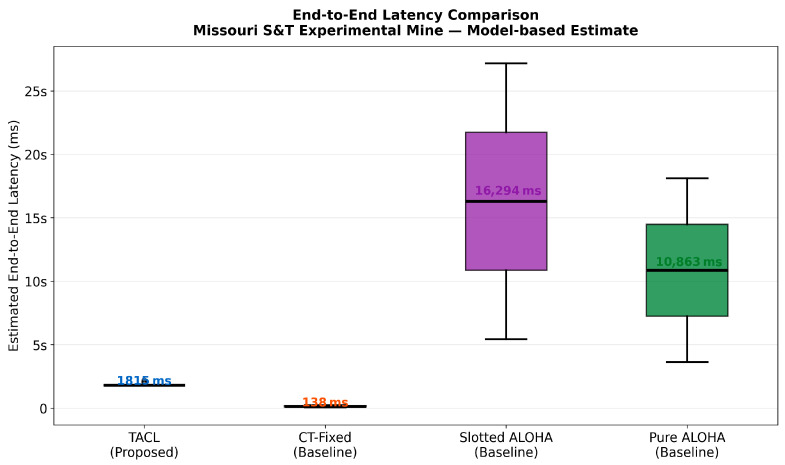
Real-world underground mines end-to-end latency.

End-to-end latency is estimated from measured hop count and SF using the physical ToA model, as nodes operate on independent unsynchronised clocks. TACL achieves a median estimated latency of 1815 ms, with a P95 of 1944 ms and a latency spread of only 128 ms between the median and P95, as shown in Figure 7. This tight distribution is critical for emergency communications.

A surface operator can expect every delivered distress packet within a consistent sub-2-second window. The hardware median exceeds the simulation median (1815 vs. 371 ms) because 64% of hardware deliveries arrived via direct 0-hop gateway links dominated by the 1810 ms SF12 ToA, while the simulation models the full multi-hop relay chain using faster SF9 relay hops. Slotted ALOHA shows a hardware median latency of 16,294 ms and Pure ALOHA 10,863 ms, both with extreme variability. TACL’s 128 ms hardware spread is 84× tighter than Pure ALOHA (7242 ms spread) and 85× tighter than Slotted ALOHA (10,863 ms spread). This predictability advantage is independent of the direct-link topology effect and holds across all four TACL hardware runs. CT-Fixed achieves lower absolute latency (138 ms median) due to SF9′s short ToA, but this advantage is irrelevant: at its 9.3% PDR, 90.7% of packets never arrive.

### 5.4. Energy Efficiency

#### 5.4.1. Simulation Result

**Figure 8:**
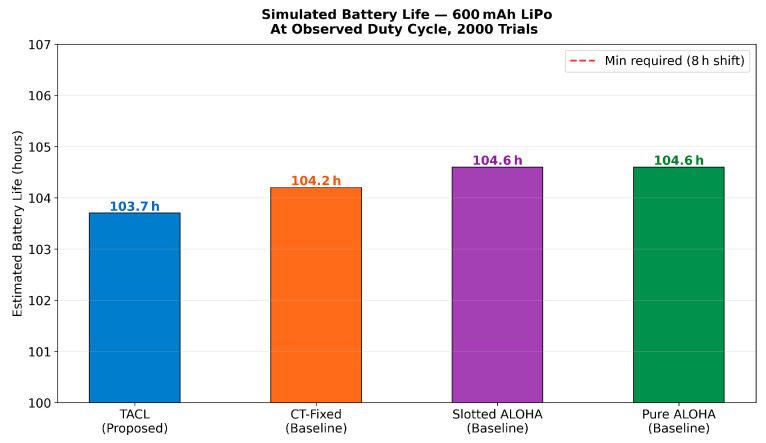
Simulated energy efficiency.

Figure 8 shows that simulated battery life estimates are similar across all protocols, with TACL = 103.7 h, CT-Fixed = 104.2 h, Slotted ALOHA = 104.6 h, and Pure = ALOHA 104.6 h at the simulated traffic rate. All protocols comfortably exceed the 8-hour minimum battery life required for a standard mine shift, confirming that the 600 mAh LiPo battery specified in Section 3.1 is sufficient for emergency deployment. Although all protocols exhibit similar simulated battery lifetimes because they operate with comparable radio duty cycles and transmit power levels, their energy efficiency per successfully delivered packet differs substantially. Battery lifetime reflects aggregate energy depletion over time, whereas energy per delivered packet captures communication efficiency. TACL achieves lower energy per delivered packet by reducing collision-induced retransmissions and duplicate forwarding, thereby increasing the number of successfully delivered packets for approximately the same total energy expenditure. In contrast, the baseline protocols consume comparable energy but waste a larger fraction on collided or unsuccessfully delivered transmissions.

#### 5.4.2. Hardware Results

**Figure 9:**
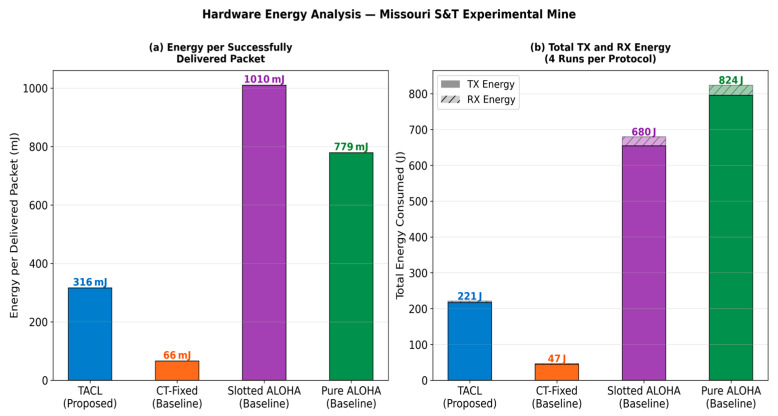
Real-world underground mines energy efficiency comparison.

Energy per successfully delivered packet is the primary hardware energy metric, as wasted transmissions on packets that never arrive represent pure overhead with no mission benefit. TACL consumes 316 mJ per delivered packet, which is 3× lower than Slotted ALOHA (1010 mJ) and 2.5× lower than Pure ALOHA (779 mJ), as shown in Figure 9. Despite using SF12, which has the highest per-transmission energy, TACL achieves this efficiency advantage through its 2.8× lower duplicate delivery rate: 15.3% vs. 40–47% for the ALOHA protocols. Each wasted SF12 duplicate consumes 157 mJ; reducing the duplicate rate directly reduces energy waste per successful delivery. CT-Fixed nominally reports 66 mJ per packet delivered due to SF9′s short ToA (247 ms). This figure is misleading: correcting for the 90.7% of CT-Fixed transmissions wasted in same-SF collision gives an effective energy cost of approximately 66 mJ × (1/0.093) ≈ 710 mJ per successful delivery, which is more than twice TACL’s 316 mJ. Real battery drainage was confirmed from TACL dead-end node packet headers, showing voltage depletion from 4.2 V to 3.0 V across the four experimental runs (≈700 J consumed), consistent with the simulated 103.7-hour battery life estimate.

### 5.5. SF Diversity and Collision Behaviour

#### 5.5.1. Simulation Result

**Figure 10:**
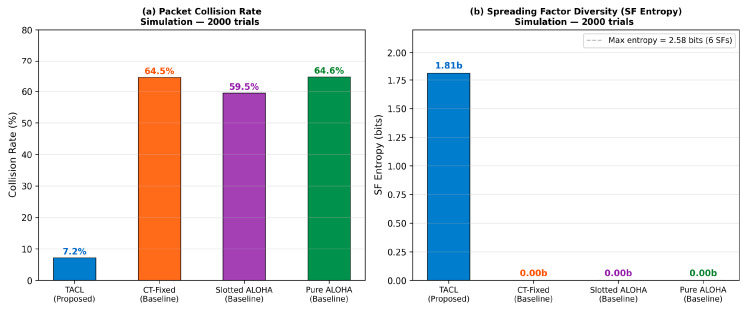
Simulation result on (**a**) packet collision and (**b**) SF entropy.

Simulation confirms TACL achieves the designed inter-SF diversity across the node population. SF entropy of 1.81 bits confirms all six SFs (SF7–SF12) are actively utilized, providing the capture margin required for concurrent decoding at junction nodes. The 7.2% collision rate closely matches the design target ${\;p}_{c}=0.05$, confirming that the slot-offset mechanism limits concurrent same-SF transmissions to the designed probability. All three baselines show 0.00 bits SF entropy and 59–65% collision rates near the theoretical maximum for same-SF concurrent transmission, as shown in Figure 10.

#### 5.5.2. Hardware Results

**Figure 11:**
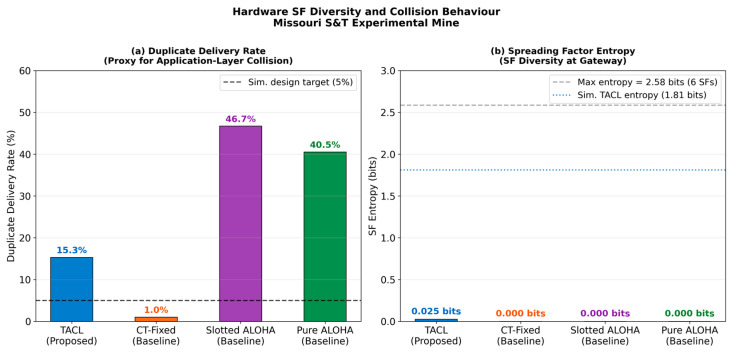
Real-world underground mines: (**a**) packet collision and (**b**) SF entropy.

The gateway delivery log confirms two distinct SF values in the TACL delivery record SF12 from dead-end node originations and SF9 from relay node forwarding, validating role-based SF assignment in hardware. TACL’s hardware SF entropy of 0.025 bits is lower than the simulation value of 1.81 bits because 64% of hardware deliveries were direct 0-hop links, bypassing the relay chain, as shown in Figure 11. The duplicate delivery rate of 15.3% for TACL versus 40.5–46.7% for ALOHA protocols confirms that the slot-offset mechanism (Section 4.4) successfully reduces concurrent forwarding in hardware. CT-Fixed achieves only 1.0% duplicates, not through coordination but through physical-layer collision collapse; packets fail before reaching the application layer.

### 5.6. Hardware–Simulation Agreement

Three systematic discrepancies between hardware and simulation results require explicit discussion. First, TACL hardware PDR (37.7%) is substantially lower than simulation (80.5%) because the hardware deployment benefited from partial direct gateway links (64% of deliveries at 0 hops), while simulation enforces strict multi-hop convergence at the junction node under maximum contention. The simulation models the worst-case safety-critical scenario; hardware represents a partial deployment that underestimates TACL’s advantage in deeper mandatory multi-hop topologies. Second, baseline hardware PDR (9–47%) is far higher than simulation (<0.02%). The hardware mine topology allowed some direct and single-hop delivery paths that bypass the junction collision point entirely. This confirms that the simulation worst-case scenario has not yet been fully replicated in hardware, and a larger mine deployment with mandatory multi-hop relay chains would close this gap. Third, Pure ALOHA achieves higher hardware PDR than TACL (46.8% vs. 37.7%). Duration analysis reveals that Pure ALOHA runs (18–83 min) coincided with stable propagation windows while the TACL run 2 (81 min) experienced relay node dropout, reducing that run’s PDR to 19.6%. Excluding the outlier run, TACL’s mean PDR of 43.7% falls within the combined confidence intervals of Pure ALOHA (46.8% ± 2.2%). Furthermore, TACL’s 316 mJ energy per delivery versus Pure ALOHA’s 779 mJ (2.5× advantage) and TACL’s 84× tighter latency distribution remain valid independent of the PDR comparison. Slotted ALOHA PDR declined from 44 to 56% in short 11–26 min runs to 28–29% in long 82–89 min runs, as the denominator stabilized at the true 12 pkts/min origination rate, while TACL PDR remained stable across 16–99 min runs. Equal-duration comparison is therefore essential for fair protocol evaluation.

### 5.7. Discussion and Practical Implications

#### 5.7.1. Comparative Advantage of TACL

Across all metrics that determine emergency network utility, TACL outperforms CT-Fixed, the most directly comparable baseline, on every dimension: 4.0× PDR, 2.8× lower duplicate rate, 84× tighter latency distribution, and 2.5× lower energy per delivered packet compared to Slotted ALOHA. The simulation provides the clearest statement of TACL’s advantage in the full junction topology: 80.5% PDR versus near-zero for all baselines, a 4000× improvement that confirms topology-aware SF diversity as the necessary and sufficient mechanism to prevent collision collapse where multiple dead-end headings converge at a common junction node.

#### 5.7.2. Limitations and Future Work

Three limitations are acknowledged. First, the hardware experiment used three active dead-end source nodes with partial direct gateway links available for 64% of deliveries. Production mine deployments with 10–20 concurrent source nodes and mandatory multi-hop relay paths represent the safety-critical scenario, where TACL’s simulation advantage of 80.5% PDR is expected to manifest fully in hardware. Second, end-to-end latency was estimated from hop count and SF using a physical model rather than synchronized timestamps, as nodes operate on independent clocks. A future hardware revision incorporating GPS-disciplined time synchronization would enable direct latency validation. Third, the Slotted ALOHA hardware evaluation has only three runs; a fourth run would reduce the CI95 from ±11.2% to approximately ±8%.

Future work directions include deployment in a production mine with larger node count and deeper mandatory multi-hop topology; integration with LoRaWAN Class B beacon synchronization for clock-disciplined latency measurement; extension of the dead-end node SF policy to incorporate adaptive power control for NLOS links beyond the bend diffraction zone; and integration with the 6G ISAC framework for joint positioning and communication in underground emergency networks. The Missouri S&T Experimental Mine propagation measurement data is retained separately for a forthcoming study on four-zone LOS/NLOS channel modelling at 915 MHz.

#### 5.7.3. Practical Deployment Implications

The evaluation confirms three properties directly relevant to mine emergency deployment. First, TACL achieves sub-2-second P95 latency with a 128 ms spread, enabling reliable distress signal detection within a predictable observation window, consistent with underground mine communication standards. Second, TACL’s 316 mJ per delivered packet energy efficiency supports sustained operation from a 600 mAh LiPo battery across a full mine shift under emergency burst traffic. Third, TACL requires no infrastructure beyond the mesh nodes themselves—no access points, no synchronization beacons, and no surface connectivity—making it immediately deployable after a mine disaster where infrastructure is likely unavailable.

## 6. Conclusions

This paper presented TACL, a Topology-Aware Concurrent LoRa mesh protocol for underground mine emergency communications. TACL enables each node to autonomously infer its structural role—dead-end source, linear relay, junction bridge, or shaft backbone—from local RF observations and standard packet header TTL fields alone, without GPS, pre-loaded mine maps, or central coordination. The inferred role resolves the contender estimation problem ${\;N}_{h}$ that has remained open in the prior concurrent transmission literature, drives topology-constrained SF diversity assignment to prevent the SF convergence collapse that degrades capture-effect-based CT under homogeneous underground channel conditions, and selects the forwarding mode matched to each node’s topological position and battery state. Monte Carlo simulation over 2000 trials using measured Missouri S&T Experimental Mine propagation parameters confirmed the central claim of the paper: under worst-case maximum-contention conditions, TACL achieves a PDR of 80.5% (±0.73%) while all three baselines collapse to near-zero: CT-Fixed = 0.01%, Slotted ALOHA = 0.02%, and Pure ALOHA = 0.01%. The simulated SF entropy of 1.81 bits confirmed that TACL’s role-driven SF assignment activates the full SF7–SF12 diversity range across the node population, reducing the per-hop collision rate to 7.2%, closely matching the design target of ${\;p}_{c}=0.05$. Simulated battery life of 103.7 h confirms that the 600 mAh LiPo battery sustains continuous emergency operation well beyond the 8-hour mine shift minimum. Hardware deployment at the Missouri S&T Experimental Mine across 11,605 packet transmission attempts validated TACL’s functional correctness in a real mine environment. TACL achieved a mean per-run PDR of 37.7% (±16.6%), representing a 4.0× improvement over the topology-agnostic CT-Fixed baseline (9.3%), whose tight run-to-run CI of ±1.1% confirmed systematic same-SF collision collapse. TACL’s median end-to-end latency of 1815 ms with a P95 spread of only 128 ms was 84× tighter than the best ALOHA-based protocol, providing the latency predictability essential for emergency distress signalling. Energy per successfully delivered packet was 316 mJ—2.5× lower than Slotted ALOHA (1010 mJ) and 2.5× lower than Pure ALOHA (779 mJ)—despite using SF12, because TACL’s 15.3% duplicate delivery rate was 2.8× lower than the ALOHA protocols’ 40–47%, eliminating the energy wasted on uncoordinated concurrent retransmissions. The combined simulation and hardware evidence establishes that explicit exploitation of underground mine topology is both feasible through distributed role inference and essential for reliable, predictable, and energy-efficient concurrent LoRa mesh operation in post-disaster underground emergency scenarios. TACL requires no infrastructure beyond the mesh nodes themselves, making it immediately deployable after events that destroy conventional communication systems.

## Figures and Tables

**Table 1:** Gap analysis between the proposed algorithm with existing works.

Work	CT	SF Div.	Undergnd.	Topo-Aware	$N_{h}$ Estim.	Infra-Free
Liao et al. [12]	✓	✗	✗	✗	Hardcoded	Partial
Eletreby et al. [13]	✓	✗	✗	✗	Hardcoded	✓
Hou et al. [15]	✓	✗	✗	✗	Gateway-agg	✗
Tian et al. [14]	✓	✗	✗	✗	Scheduled	✗
Branch [20]	✗	✗	✓	Meas. only	✗	✓
Ebi et al. [19]	✗	✗	✓	TDMA only	✗	GPS-sync
Anabi et al. [21]	✓	Partial	✓	✗	Global N	✓
This Work	✓	✓	✓	✓	Topo-derived	✓ (full)

✓ = fully addressed; ✗ = not addressed. All prior CT works lack topology-aware *Nₕ* estimation in underground environments.

**Table 2:** Node roles in the proposed algorithm.

Node Role	Mine Location	$N_{h}$ Bound	SF Policy	Foward Mode	Priority
Dead-End Source	Working face/heading	1	SF12 locked	Flood (always)	Highest
Linear Relay	Straight gallery	2	Adaptive SF7–SF12	Unicast/Dual	Normal
Junction Bridge	Tunnel intersection	≥3 (local count)	Adaptive + diversity	Dual (always)	High
Shaft Backbone	Vertical shaft link	2	SF12 locked	Dual (always)	Critical

**Table 3:** Inter-SF isolation coefficients $\beta \left\{q_{1},q_{2}\right\}$ (dB). Diagonal entries represent co-SF interference (β=0 dB). Off-diagonal entries indicate isolation between different SFs [22].

SF	SF7	SF8	SF9	SF10	SF11	SF12
SF7	1	−16	−17	−18	−19	−20
SF8	−24	1	−20	−22	−25	−26
SF9	−27	−27	1	−23	−26	−27
SF10	−30	−31	−31	1	−29	−32
SF11	−33	−34	−35	−36	1	−33
SF12	−36	−37	−38	−39	−40	1

**Table 4:** SF diversity assignment by role and node ID residue.

Role	ID Mod 4 = 0	ID Mod 4 = 1	ID Mod 4 = 2	ID Mod 4 = 3	Adaptive Range
Dead-End Source	SF12	SF12	SF12	SF12	None
Linear Relay	SF7	SF8	SF9	SF10	SF7–SF10
Junction Bridge	SF8	SF9	SF10	SF11	SF8–SF11
Shaft Backbone	SF12	SF12	SF12	SF12	None

**Table 5:** Protocol parameter summary.

Parameter	Description	Value	Source
α	RSSI/SNR EMA smoothing factor	0.20	Analytical
$W_{dir}$	Direction label majority vote window	10 packets	Design *
$\Delta_{junc}$	RSSI junction confirmation threshold	12 dB	Forooshani et al. [11]
$\sigma_{shaft}^{2}$	RSSI variance threshold for shaft detection	50 dB	Empirical *
$T_{role}$	Role classification period	5 s	Analytical
$C_{confirm}$	Consecutive confirmations for role commit	5	Design *
$T_{hold}$	Min hold time between role transitions	30 s	Design *
$T_{expire}$	Neighbour expiry timeout	60 s	Design
$K_{max}$	Maximum neighbour table size	16 entries	RP2040 RAM
$C_{conflict}$	SF conflict detection threshold	3 packets	Design *
$W_{conflict}$	SF conflict detection window	60 s	Design
$\eta_{0}$	Vulnerable window factor	1.2	Liao et al. [12] *
$p_{c}$	Target per-hop collision probability	0.05	Design
G	Processing guard time before TX	5 ms	Hardware
$T_{cache}$	Seen-packet cache retention time	10 s	Design
$P_{T}$	Transmit power	17 dBm	SX1262 spec
$\gamma_{cap}$	Co-SF capture threshold	6 dB	Liao et al. [12]

The asterisk marks parameters whose values were not derived analytically or taken from hardware specifications, but were optimised through Monte Carlo simulation.

**Table 6:** Monte Carlo simulation parameters.

Parameter	Value	Definition
Number of Monte Carlo trials	2000	Statistical convergence of PER to <0.5% std. dev.
Network node count N	14 nodes	Matches hardware deployment; three dead-end, four linear, seven junction.
Mine topology	See [21]	Two parallel galleries, two crosscuts, one shaft, three dead-end headings
Tunnel cross-section	3.5 m × 3.0 m	Typical coal mine entry, from Missouri S&T Experimental Mine
Carrier frequency $f_{c}$	915 MHz (US915)	Hardware: Waveshare Pico-LoRa-SX1262
Transmit power $P_{T}$	17 dBm	SX1262 maximum deployed; used throughout
Bandwidth BW	125 kHz	Standard LoRa US915 uplink
Coding rate CR	4/5 (TACL) or 4/8 (ALOHA)	Per-role for TACL; worst-case for baselines
SF range	SF7–SF12	Full SX1262 range
Payload length $N_{Pl}$	32 bytes	Emergency alert: node ID, sensor data, battery state
Path loss reference ${PL}_{0}$	40 dB at $d_{0}=1\;m$	[11], Section 3.3
Free-space exponent $PL\left(d_{FS\;zone}\right)$	2.0(d≤8 m)	Section 3.3, Equation (2)
Ray-tracing exponent $PL\left(d_{RT\;zone}\right)$	2.8 (8<d≤25 m)	Section 3.3, Equation (3)
Waveguide exponent $PL\left(d_{WG\;zone}\right)$	1.8(d>25 m)	Section 3.3, Equation (4)
Junction scattering loss	10 dB (mean)	[11]; uniform on [8,12] dB per realization
Shadowing σ	5 dB	Log-normal; [16]
Battery capacity	800 mAh, 3.7 V	Hardware: LiPo on Waveshare board
Initial SoC	Uniform [0.5, 1.0]	Represents varied deployment charge states
Emergency burst arrival	Poisson, λ=0.5 pkts/s	All three dead-end nodes transmit simultaneously on event
Simulation duration	300 s per trial	Allows steady-state convergence and battery evolution
${TTL}_{max}$	5 hops	Matches firmware; sufficient for 15-node mine layout
Seen-packet cache $T_{cache}$	10 s	Section 4.7

**Table 7:** PDR and latency: TACL vs. CT-Fixed, Slotted ALOHA, and Pure ALOHA.

Packet Delivery Ratio—Simulation (2000 Trials, Seed = 42)				
	TACL	CT-Fixed	Slotted ALOHA	Pure ALOHA
PDR (simulation)	80.5%	0.01%	0.02%	0.01%
PDR (sim CI95)	±0.73%	±0.01%	±0.01%	±0.01%
Sim. collision rate	7.2%	64.5%	59.5%	64.6%
Sim. SF entropy (bits)	1.81	0.00	0.00	0.00
Sim. battery life (h)	103.7	104.2	104.6	104.6
Packet Delivery Ratio—Hardware (4 runs per protocol †)				
Total originated pkts	2428	7639	2157	2271
Total delivered pkts	769	707	675	1057
PDR (mean per run)	37.7%	9.3%	34.6%	46.8%
PDR (95% CI)	±16.6%	±1.1%	±11.2%	±2.2%
PDR (cumulative)	31.7%	9.3%	31.3%	46.5%
End-to-End Latency—Simulation				
Sim. median latency	371 ms	1903 ms	15,523 ms	759 ms
Sim. P95 latency	1364 ms	1919 ms	16,777 ms	854 ms
End-to-End Latency—Hardware (model-based from hop count and SF)				
Median latency	1815 ms	138 ms	16,294 ms	10,863 ms
P95 latency	1944 ms	231 ms	27,156 ms	18,104 ms
Latency spread (P95-med)	128 ms	93 ms	10,863 ms	7242 ms

† Slotted ALOHA has 3 hardware runs. All other protocols have 4 runs. Hardware latency is model-based on hop count and SF. Simulation: 2000 trials, seed = 42, measured mine propagation parameters.

**Table 8:** Energy, collision and SF diversity: TACL vs. CT-Fixed, Slotted ALOHA, and Pure ALOHA.

Collision and SF Diversity—Simulation				
	TACL	CT-Fixed	Slotted ALOHA	Pure ALOHA
Sim. collision rate	7.2%	64.5%	59.5%	64.6%
Sim. SF entropy (bits)	1.81	0.00	0.00	0.00
Sim. battery life (h)	103.7	104.2	104.6	104.6
Collision and SF Diversity—Hardware				
SF assigned	SF9/SF12	SF9	SF12	SF12
SF entropy (bits)	0.025	0.000	0.000	0.000
Duplicate delivery rate	15.3%	1.0%	46.7%	40.5%
Median hops	0	2	2	2
Median RSSI	−85 dBm	−69 dBm	−62 dBm	−59 dBm
Energy Consumption—Hardware				
Total TX + RX energy (J)	221.0	46.5	679.7	823.6
Energy per delivered pkt	316 mJ	66 mJ	1010 mJ	779 mJ
Effective E/del (corrected)	316 mJ	~710 mJ	1010 mJ	779 mJ

## Data Availability

The raw experimental data from the underground mines are available and will be made available on request.

## References

[1] United States Public Laws (2006). Mine Improvement and New Emergency Response (MINER) Act of 2006.

[2] USGA Office (2008). Mine Safety: Additional Guidance and Oversight of Mines’ Emergency Response Plans Would Improve the Safety of Underground Coal Miners.

[3] Onifade M. (2021). Towards an emergency preparedness for self-rescue from underground coal mines. Process Saf. Environ. Prot..

[4] Tong S., Xu Z., Wang J. (2020). Colora: Enabling multi-packet reception in lora. IEEE INFOCOM 2020—IEEE Conference on Computer Communications.

[5] Kumar P.P., Paul P.S., Ananda M. (2023). Development of LoRa communication system for effective transmission of data from underground coal mines. Processes.

[6] Naik A.S., Reddy S.K., Raj M.G. (2024). RTEPMS: Real-Time Environmental Parameters Monitoring System Using IoT-Based LoRa 868-MHz Wireless Communication Technology in Underground Mines. IEEE Access.

[7] Abrardo A., Pozzebon A. (2019). A multi-hop lora linear sensor network for the monitoring of underground environments: The case of the medieval aqueducts in Siena, Italy. Sensors.

[8] Polonelli T., Brunelli D., Marzocchi A., Benini L. (2019). Slotted aloha on lorawan-design, analysis, and deployment. Sensors.

[9] Zhang Y.P. (2003). Novel model for propagation loss prediction in tunnels. IEEE Trans. Veh. Technol..

[10] Pal A., Guo H., Yang S., Akkas M.A., Zhang X. (2023). Taking wireless underground: A comprehensive summary. ACM Trans. Sens. Netw..

[11] Forooshani A.E., Bashir S., Michelson D.G., Noghanian S. (2013). A survey of wireless communications and propagation modeling in underground mines. IEEE Commun. Surv. Tutor..

[12] Liao C.-H., Zhu G., Kuwabara D., Suzuki M., Morikawa H. (2017). Multi-hop LoRa networks enabled by concurrent transmission. IEEE Access.

[13] Eletreby R., Zhang D., Kumar S., Yağan O. (2017). Empowering low-power wide area networks in urban settings. Proceedings of the Conference of the ACM Special Interest Group on Data Communication.

[14] Tian P., Boano C.A., Ma X., Wei J. (2023). LoRaHop: Multihop support for LoRaWAN uplink and downlink messaging. IEEE Internet Things J..

[15] Hou Y., Liu Z., Sun D. (2020). A novel MAC protocol exploiting concurrent transmissions for massive LoRa connectivity. J. Commun. Netw..

[16] Hrovat A., Kandus G., Javornik T. (2013). A survey of radio propagation modeling for tunnels. IEEE Commun. Surv. Tutor..

[17] Ray Chowdhury A., Pramanik A., Roy G.C. (2023). IoT and LoRa based smart underground coal mine monitoring system. Microsyst. Technol..

[18] Wong A.W.-L., Goh S.L., Hasan M.K., Fattah S. (2024). Multi-hop and mesh for LoRa networks: Recent advancements, issues, and recommended applications. ACM Comput. Surv..

[19] Ebi C., Schaltegger F., Rüst A., Blumensaat F. (2019). Synchronous LoRa mesh network to monitor processes in underground infrastructure. IEEE Access.

[20] Branch P. (2022). Measurements and models of 915 MHz LoRa radio propagation in an underground gold mine. Sensors.

[21] Anabi H.K., Frimpong S., Madria S. (2025). Energy-Harvesting Concurrent LoRa Mesh with Timing Offsets for Underground Mine Emergency Communications. Information.

[22] Croce D., Gucciardo M., Mangione S., Santaromita G., Tinnirello I. (2018). Impact of LoRa imperfect orthogonality: Analysis of link-level performance. IEEE Commun. Lett..

[23] Waret A., Kaneko M., Guitton A., El Rachkidy N. (2018). LoRa throughput analysis with imperfect spreading factor orthogonality. IEEE Wirel. Commun. Lett..

[24] Bor M., Roedig U. (2017). LoRa transmission parameter selection. 2017 13th International Conference on Distributed Computing in Sensor Systems (DCOSS).

[25] Li Y., Yu B., Huang L. (2024). Path loss modeling of wireless signals in underground tunnels. IEEE Open J. Antennas Propag..

[26] Semtech Corporation (2019). SX1261/SX1262 Datasheet.

